# Bisphenol A and Its Analogues Activate Human Pregnane X Receptor

**DOI:** 10.1289/ehp.1104426

**Published:** 2012-01-03

**Authors:** Yipeng Sui, Ni Ai, Se-Hyung Park, Jennifer Rios-Pilier, Jordan T. Perkins, William J. Welsh, Changcheng Zhou

**Affiliations:** 1 Graduate Center for Nutritional Sciences, University of Kentucky, Lexington, Kentucky, USA; 2 Department of Pharmacology, Robert Wood Johnson Medical School, University of Medicine and Dentistry of New Jersey, Piscataway, New Jersey, USA

**Keywords:** BPA, BPB, endocrine-disrupting chemicals, PXR, SXR

## Abstract

Background: Bisphenol A (BPA) is a base chemical used extensively in many consumer products. BPA and its analogues are present in environmental and human samples. Many endocrine-disrupting chemicals, including BPA, have been shown to activate the pregnane X receptor (PXR), a nuclear receptor that functions as a master regulator of xenobiotic metabolism. However, the detailed mechanism by which these chemicals activate PXR remains unknown.

Objective: We investigated the mechanism by which BPA interacts with and activates PXR and examined selected BPA analogues to determine whether they bind to and activate PXR.

Methods: Cell-based reporter assays, *in silico* ligand–PXR docking studies, and site-directed mutagenesis were combined to study the interaction between BPA and PXR. We also investigated the influence of BPA and its analogues on the regulation of PXR target genes in human LS180 cells.

Results: We found that BPA and several of its analogues are potent agonists for human PXR (hPXR) but do not affect mouse PXR activity. We identified key residues within hPXR’s ligand-binding pocket that constitute points of interaction with BPA. We also deduced the structural requirements of BPA analogues that activate hPXR. BPA and its analogues can also induce PXR target gene expression in human LS180 cells.

Conclusions: The present study advances our understanding of the mechanism by which BPA interacts with and activates human PXR. Activation of PXR by BPA may explain some of the adverse effects of BPA in humans.

There is growing concern about the possible adverse health effects posed by endocrine-disrupting chemicals (EDCs), which can interfere with an organism’s complex endocrine signaling mechanisms and have deleterious consequences (Diamanti-Kandarakis et al. 2009). One EDC in particular, bisphenol A (BPA), has attracted considerable attention and controversy. BPA is among the world’s highest production volume chemicals, with > 8 billion pounds produced each year (Vandenberg et al. 2010). BPA, a base chemical used extensively in polycarbonate plastics, is present in many consumer products (Bondesson et al. 2009; vom Saal et al. 2007). More than 80 biomonitoring studies indicate that human exposure to BPA is ubiquitous globally, and most of the U.S. population (> 90%) is exposed to BPA (Vandenberg et al. 2010).

BPA has been detected in human blood, urine, tissues, and other fluids, and higher levels have been detected in infants and children than in adults (Vandenberg et al. 2010). Numerous animal studies indicate that even low-level exposures to BPA may cause adverse health effects in humans (Bondesson et al. 2009; Vandenberg et al. 2010; vom Saal et al. 2007), and higher BPA exposure has been associated with diabetes and cardiovascular disease in the general adult U.S. population (Lang et al. 2008; Melzer et al. 2010). Despite evidence of adverse effects of BPA in humans, the underlying mechanisms remain elusive. BPA, regarded as a xenoestrogen, is a weak agonist of the estrogen receptor (ER) (Bondesson et al. 2009). Nevertheless, doubts remain whether BPA exerts adverse estrogenic effects in animals and humans (Sharpe 2010; Vandenberg et al. 2009).

Many EDCs, including organochlorine and organophosphate pesticides, alkylphenols, phthalates, and polychlorinated biphenyls (PCBs) activate another nuclear receptor, the pregnane X receptor (PXR; also known as steroid and xenobiotic receptor, or SXR) (Zhou et al. 2009b). PXR is a xenobiotic sensor that regulates clearance via induction of genes involved in drug and xenobiotic metabolism (Zhou et al. 2009b). Recently, PXR has been implicated in lipid homeostasis, atherosclerosis, and carcinogenesis (Sui et al. 2011; Wang et al. 2011; Zhou C et al. 2009a; Zhou J et al. 2006).

BPA can activate human PXR (hPXR) and induce target gene expression in human cells (Tabb et al. 2004; Takeshita et al. 2001). However, the detailed mechanism by which BPA exerts these effects remains unknown. Numerous BPA analogues and derivatives, including bisphenol B (BPB), bisphenol F (BPF), bisphenol S (BPS), brominated derivative bisphenols [e.g., tetrabromobisphenol A (TBBPA)], and chlorinated bisphenols [e.g., tetrachlorobisphenol A (TCBPA)], are used in commodity products (Cobellis et al. 2009; Shi et al. 2009). Although BPB, TBBPA, and TCBPA have been detected in human serum and tissues (Cobellis et al. 2009; Fernandez et al. 2007; Shi et al. 2009), it is not known if these environmental chemicals activate PXR in a manner similar to that of BPA.

Here we report that BPA is a potent agonist for hPXR but not for mouse or rat PXR (mPXR and rPXR, respectively). Cell-based reporter assays, *in silico* ligand–PXR docking studies, and site-directed mutagenesis were combined to study the interaction between BPA and PXR. Several BPA analogues also functioned as hPXR agonists. Moreover, we observed that combinations of BPA and certain analogues work synergistically to activate PXR and induce PXR target gene expression in human LS180 cells. These studies reveal that hPXR is a target of BPA and suggest that some effects of BPA in humans may arise in part from PXR activation.

## Materials and Methods

*Reagents and plasmids.* BPA, pregnenolone 16α-carbonitrile (PCN), rifampicin (RIF), and 2,2-diphenylpropane (DPP) were purchased from Sigma-Aldrich (St. Louis, MO). BPA β-d-glucuronide was purchased from Santa Cruz Biotechnology (Santa Cruz, CA). All other BPA analogues were purchased from TCI America (Portland, OR). All chemicals were dissolved in dimethyl sulfoxide (DMSO). hPXR and mPXR expression vectors; GAL4 DNA-binding domain (DBD)-linked nuclear receptor ligand-binding domain (LBD) vectors; VP16-hPXR, GAL4-NCoR, GAL4-SMRT, GAL4-SRC1, GAL4-PBP, and CMX–β-galactosidase expression vectors; and hPXR reporter (CYP3A4XREM-luciferase), mPXR reporter [(CYP3A2)_3_-luciferase], and GAL4 reporter (MH100-luciferase) have been described previously (Blumberg et al. 1998; Tabb et al. 2004; Zhou et al. 2007).

*Site-directed mutagenesis.* hPXR full-length plasmid was used as a wild-type template to generate a series of mutant plasmids by using the QuikChange II Site-Directed Mutagenesis Kit (Agilent, Santa Clara, CA) according to the manufacturer-supplied protocol. The primers used for mutant generation are listed in Supplemental Material, Table 1 (http://dx.doi.org/10.1289/ehp.1104426).

*Cell culture and transfections.* The human hepatic cell line HepG2 and intestine epithelial cell line LS180 were obtained from American Type Culture Collection (Manassas, VA). Cells were transfected using FuGENE 6 (Roche Diagnostics, Indianapolis, IN), and luciferase and β-galactosidase activities were determined as previously described (Tabb et al. 2004; Zhou et al. 2007). For the mammalian two-hybrid assays, HepG2 cells were transfected with GAL4 reporter, VP16-hPXR, and GAL4-SRC1, GAL4-PBP, GAL4-SMRT, or GAL4-NCoR plasmids. The cells were then treated with compounds at the indicated concentration. Cytotoxicity was assessed using the 3-(4,5-dimethythiazol-2-yl)-2,5-diphenyltetrazolium bromide (MTT) assay (Mosmann 1983).

*RNA isolation and quantitative real-time polymerase chain reaction (QPCR) analysis.* Total RNA was isolated from LS180 cells using TRIzol reagent (Life Technologies, Carlsbad, CA) according to the manufacturer-supplied protocol. QPCR was performed as described previously (Sui et al. 2011). The primers are listed in Supplemental Material, Table 1 (http://dx.doi.org/10.1289/ehp.1104426).

*Computational docking studies.* The structural coordinates of the tethered hPXR, linker, steroid receptor coactivator 1 (PXR/SRC-1) were retrieved from the RSCB Protein Data Bank entry 3HVL (RSCB Protein Data Bank 2012; Wang et al. 2008). The larger PXR fragment of chain A, Gly142-Glu458, was extracted for molecular modeling using MOE 2010 software (Chemical Computing Group, Montreal, Quebec, Canada), and for ligand-receptor docking studies using GOLD software (version 5.0) (Payne and Glen 1993). Water molecules, salt ions, ligand (SR12813), and coreceptor fragments were deleted. After the addition of hydrogen atoms and assigning of the AMBER99 force-field charges to the protein, the hydrogen atomic positions were allowed to relax (Wang et al. 2000). The resulting protein structural coordinates were saved in Tripos mol2 format and used later for GOLD docking.

The ligands were docked to the 3HVL chain A using semiflexible docking whereby the ligand has full conformational flexibility and the hydroxyl groups of designated protein side chains in the binding pocket can rotate to optimize hydrogen bond contacts. Each ligand was docked 50 independent times. The binding pocket was defined as all the atoms within an 8-Å radius around the bound ligand, SR12813. For mutagenesis study, we performed local energy minimization after each *in silico* mutation and compared the backbone root-mean-square deviation (RMSD) of the wild-type and mutated folds in the region of the PXR binding pocket. The calculated RMSD was < 1.0 Å in all cases, so alteration in protein folds was minimal.

*Statistical analysis.* Statistical analysis of the luciferase reporter assays was performed using a two-sample, two-tailed Student’s *t*-test, with *p* < 0.05 regarded as significant. One-way analysis of variance was used when multiple comparisons were made, followed by Dunnett’s *t*-test for multiple comparisons with controls.

## Results

**Figure 1:**
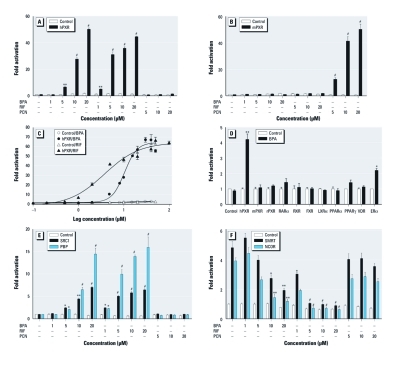
Species-specific activation of PXR by BPA. (*A* and *B*) HepG2 cells were transfected with full-length hPXR together with a hPXR reporter (CYP3A4‑luc; *A*) or full-length mPXR together with an mPXR reporter [(CYP3A2)_3_-luc); *B*] and CMX–β-galactosidase control plasmid. Cells were then treated with DMSO (control), BPA, RIF (hPXR ligand), or PCN (mPXR ligand) for 24 hr. (*C*) HepG2 cells were co-transfected with hPXR together with CYP3A4-luc reporter and CMX–β-galactosidase plasmid; cells were then treated with BPA or RIF at concentrations 0.1–100 μM for 24 hr. (*D*) HepG2 cells were co-transfected with a GAL4 reporter and a series of GAL4 constructs in which the GAL4 DBD is linked to the indicated nuclear receptor LBD; cells were treated with DMSO or 5 μM BPA for 24 hr. Abbreviations: FXR, farnesoid X receptor; LXRα, liver X receptor-α; PPARα and PPARγ, peroxisome proliferator-activated receptor-α and -γ; RARα, retinoid acid receptor-α; RXR, retinoid X receptor; VDR, vitamin D receptor. (*E* and *F*) HepG2 cells were transfected with a GAL4 reporter and VP16-hPXR, as well as expression vector for GAL4 DBD or GAL4 DBD linked to the receptor interaction domains of the indicated PXR coactivators (GAL4-SRC1 and GAL4-PBP; *E*) or corepressor (GAL4-SMRT and GAL4-NCoR; *F*). Cells were then treated with DMSO, BPA, RIF, or PCN for 24 hr. Error bars indicate SEM. *p < 0.05, **p < 0.01, and ^#^*p* < 0.001 compared with control (*n* = 3–5 per group).

*Species-specific activation of PXR by BPA.* In mammals, PXR exhibits considerable differences in its pharmacology, in part because of the remarkable divergence in its LBD across species (Blumberg et al. 1998; Zhou et al. 2009b). We first tested the ability of BPA to activate hPXR or mPXR using transfection assays. The potent species-specific PXR ligands rifampicin (RIF) and pregnenolone 16α-carbonitrile (PCN) were used as the positive control for hPXR and mPXR, respectively. BPA strongly induced hPXR reporter activities in a dose-dependent manner (Figure 1A) but had no effect on mPXR activity (Figure 1B). BPA elicited no toxicity to the cells at tested concentrations as revealed by the MTT assay [Supplemental Material, Figure 1 (http://dx.doi.org/10.1289/ehp.1104426)]. Dose–response analysis indicated that the half-maximal effective concentration (EC_50_) for BPA activation of hPXR-mediated cytochrome P450 3A4 (CYP3A4) promoter activity was about 9 μM (Figure 1C).

To determine whether BPA acts specifically on hPXR, we evaluated the ability of BPA to activate a number of other nuclear receptors, including rPXR, human retinoid acid receptor (RAR)α, retinoid X receptor (RXR), farnesoid X receptor (FXR), liver X receptor (LXR)α, peroxisome proliferator-activated receptor (PPAR)α, PPARγ, vitamin D receptor (VDR), and ERα (Figure 1D). BPA had little, if any, effect on activation of these other nuclear receptors, with the exception of ERα (Figure 1D); therefore, we focused our attention on BPA-mediated PXR agonism. These data suggest that BPA is a species-selective agonist of hPXR, but not mPXR and rPXR, similar to the species selectivity of RIF for hPXR compared with mPXR and rPXR.

Nuclear receptor coregulators play critical roles in nuclear receptor activation. We used the mammalian two-hybrid assay to evaluate whether BPA affects the interaction of PXR with coactivators or corepressors. Both RIF and BPA strongly promoted the specific interaction of steroid receptor coactivator-1 (SRC1) and peroxisome proliferator–activated receptor-binding protein (PBP) in a dose-dependent manner (Figure 1E). Unliganded hPXR was able to interact with the corepressors nuclear receptor corepressor (NCoR) and silencing mediator of retinoid and thyroid hormone (SMRT; Figure 1F). Similar to RIF, BPA promotes the dissociation of hPXR from NCoR or SMRT (Figure 1F). In contrast, PCN had no effect on the interactions of hPXR with the coactivators (Figure 1E) or corepressors (Figure 1F). Binding of BPA to hPXR inhibits PXR/corepressor interaction and promotes PXR coactivator recruitment, thereby inducing hPXR transcriptional activation in a concentration-dependent manner.

**Figure 2:**
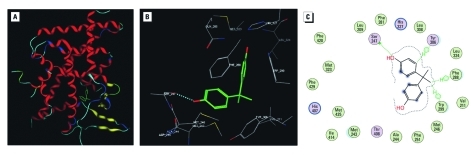
Computational docking of BPA to the ligand-binding pocket of the hPXR apoprotein X-ray crystal structure (3hvl.pdb). (*A*) The BPA–hPXR complex. The PXR LBD is shown in ribbon presentation (red, helix; yellow, strand); BPA (phenyl ring, green) occupies only a small portion of the large ligand-binding pocket of PXR. (*B*) BPA forms a hydrogen bond (light blue dotted line) with Ser247 and van der Waals contacts with several other hydrophobic residues, particularly Phe288, Trp299, and Tyr306. (*C*) An interaction map of BPA and hPXR, which reveals that BPA occupies only a small portion of the large, flexible PXR ligand-binding pocket.

*Computational docking and modeling studies.* We implemented a structure-based computational approach to investigate the potential interaction pattern between BPA and PXR. BPA was docked into the X-ray crystal structure of hPXR–SR12813 cocomplex after removal of the ligand (Wang et al. 2008). After 50 independent docking exercises, a single orientation was identified for BPA in the ligand-binding pocket of hPXR. BPA forms a hydrogen bond with one PXR side chain and additional interactions with 10 other residues (Figure 2). BPA can position either one of its *para* hydroxyl groups on the phenol rings and can orient itself to form a strong O–H–O hydrogen bond (calculated 2.4 Å) with the side chain of Ser247. This dominant interaction appears to secure the ligand in a localized region of the ligand-binding pocket, leaving a significant portion of the cavity unoccupied. Nonpolar contacts also play a key role in stabilizing BPA within the ligand-binding pocket. The Ser247 hydrogen-bonded phenolic ring of BPA appears to form a 3.4-Å edge-to-face contact with the side chain of Tyr306 and a 4.0-Å hydrophobic contact with Phe288, and is surrounded by the hydrophobic side chains of Met243 and Met246. The other phenol ring of BPA is stabilized by π–π stacking interactions (3.6 Å) with the side chain of Trp299 and hydrophobic contacts (4.1 Å) with Leu324. The two methyl groups in the linker engage in hydrophobic interactions with Val211 and Trp299.

Based on visual analysis, the docked BPA ligand lacks direct contact with activation function 2 (AF-2) helix (αAF) of the PXR AF-2 surface. However, Ser247, which forms hydrogen bonds with BPA, is located in close proximity to the AF-2 residues Met425 and Phe429. These indirect interactions between BPA and the AF-2 surface may stabilize the active AF-2 conformation of the receptor and contribute to the agonistic activity of BPA on hPXR (Figure 2B,C).

Regarding the selectivity of BPA for hPXR over mPXR, sequence alignment of the LBDs of mPXR and hPXR revealed that the most divergent region of the PXR ligand-binding pocket for these two species is located at the conformationally flexible base of the cavity. This variable region includes two additional β-strands unique to PXR and a flexible loop (residue 308–321). Leu308 in hPXR, which lies in the C-terminus of the β4-strand, is replaced by Phe in mPXR. Given the greater size and rigidity of Phe compared with Leu, one might surmise from this substitution alone the absence of BPA agonistic activity in mPXR. In this case, Leu308 may play a key structural role in specific activation of hPXR by BPA.

**Figure 3:**
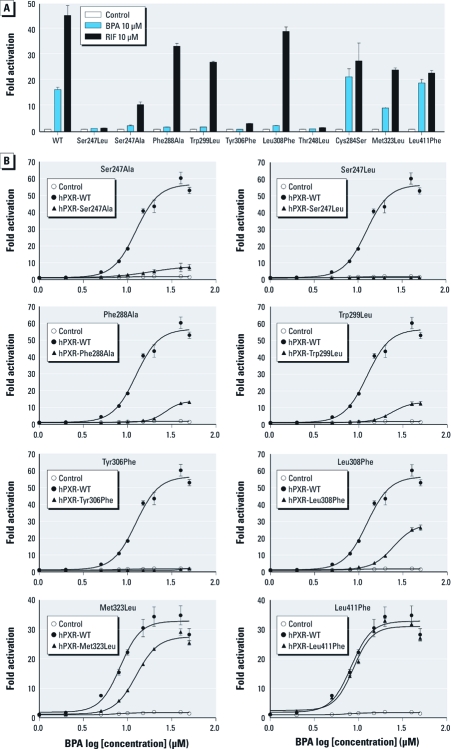
Key residues of PXR LBD are required for BPA’s agonistic activity. (*A*) Agonistic activity of BPA and RIF in HepG2 cells co-transfected with a full-length hPXR wild-type (WT) plasmid or mutant hPXR plasmids, together with CYP3A4-luc reporter and CMX–β-galactosidase plasmid and treated with control medium (medium plus DMSO) or medium containing 10 μM BPA or RIF for 24 hr. (*B*) Dose–response assay showing agonistic activity of BPA in HepG2 cells transiently transfected with a full-length hPXR plasmid or mutant hPXR plasmids, as well as CYP3A4-luciferase reporter and CMX–β-galactosidase plasmid, followed by the incubation with control medium or medium containing BPA. Error bars indicate SEM.

*Key LBD residues of PXR are required for BPA’s agonistic activity.* Guided by the results from the docking analysis, we mutated the key amino acids predicted as responsible for BPA’s agonistic activity: Ser247, Phe288, Trp299, Tyr306, and Leu308. Thr248, a key amino acid known to be important for PXR/coactivator interaction, was mutated as the positive control in this study. For negative controls, we used residues within PXR’s ligand-binding pocket predicted by the docking analysis not to interact with BPA: Cys284, Met323, and Leu411. As shown in Figure 3A, Ser247Leu, Phe288Ala, Trp299Leu, Tyr306Phe, and Leu308Phe mutations completely blocked the agonistic activity of BPA. In addition, Phe288Ala, Trp299Leu, and Leu308Phe mutations abolished BPA’s agonistic activity but had little effect on the activity of RIF. As expected, Thr248Leu mutation abolished the activity of both BPA and RIF, whereas Cys284Ser, Met323Leu, and Leu411Phe had negligible effects on BPA. Interestingly, Ser247Leu blocked the activity of both BPA and RIF, although Ser247Ala partially restored the activity of RIF.

In the dose–response assays for several mutants (Figure 3B), we observed that the Phe288Ala, Trp299Leu, and Leu308Phe PXR mutants were weakly activated by BPA at high concentrations (e.g., 25 or 50 μM); however, the Ser247Ala, Ser247Leu,, and Tyr306Phe mutants were inactive even at these concentrations. As for the negative controls, Leu411Phe did not affect BPA’s agonistic activity, whereas Met323Leu diminished activity only slightly. In summary, our site-directed mutagenesis analysis confirmed predictions from the docking study and revealed specific key residues within the ligand-binding pocket of PXR that play a significant role in the agonistic effects of BPA.

**Figure 4:**
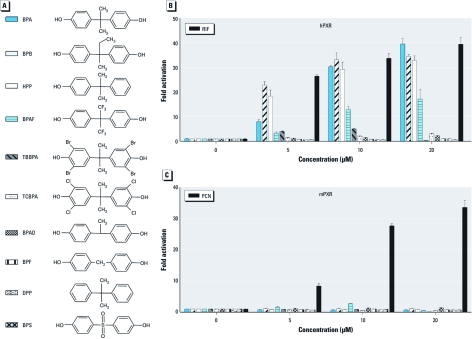
BPA analogues activate hPXR. (*A*) Chemical structure of BPA analogues. (*B* and *C*) HepG2 cells were transfected with (*B*) full-length hPXR together with CYP3A4-luc reporter or (*C*) full-length mPXR together with (CYP3A2)_3_-luc reporter and CMX–β-galactosidase control plasmid. Cells were treated with DMSO (control) or BPA analogues for 24 hr. RIF (*B*) and PCN (*C*) were used as positive controls. Error bars indicate SEM.

*Identification of BPA analogues as hPXR agonists.* In addition to BPA, many BPA analogues and derivatives are also present in the environment and found in human samples. Consequently, we next tested a series of structural analogues of BPA to determine whether they can also activate PXR (Figure 4A). We found that several of these BPA analogues, including BPB, 2-(4´-hydroxyphenyl)-2-phenylpropane (HPP), and bisphenol AF (BPAF), could activate hPXR in a dose-dependent manner (Figure 4B). BPB and HPP were more potent than BPA as hPXR agonists at a low concentration (5 μM), and had comparable agonistic effects at high concentrations (10 and 25 μM). BPAF was a relatively weak PXR agonist compared with BPA. Similar to BPA, none of them activated mPXR (Figure 4C). The other BPA analogues tested, including TCBPA, TBBPA, BPF, BPS, bisphenol AD (BPAD), and DPP, did not activate PXR. Interestingly, BPA-glucuronide, the major BPA metabolite in animals and humans, did not activate PXR [Supplemental Material, Figure 2 (http://dx.doi.org/10.1289/ehp.1104426)]. The glucuronide moiety may preclude BPA from interacting with PXR.

These results provide sufficient information for us to propose a rudimentary structure–activity relationship for PXR agonistic activity of BPA and its analogues. Specifically, the minimum requirement for hPXR agonistic activity is the presence of at least one *para* phenolic group. This feature is exemplified by the agonistic activity of BPA and HPP but not DPP. Furthermore, the two methyl groups in the linker between the phenolic rings are critical for BPA’s activity; losing one methyl group (BPAD) or both methyl groups (BPF) or replacing the C(CH_3_)_2_ linker of BPA by a sulfone group SO_2_—as in BPS—abolished agonistic effect. However, the analogue in which each of the CH_3_ groups in the linker are replaced by CF_3_ (BPAF) retained partial agonistic activity. Interestingly, the inability of TCBPA and TBBPA to activate hPXR suggests that the presence of the bromine or chlorine atoms proximal to the phenolic –OH weakens or disrupts on the O–H–O hydrogen bond with Ser247. This influence of the halogen atoms may be caused by electronic (induction, resonance) effects, steric effects, or both.

**Figure 5:**
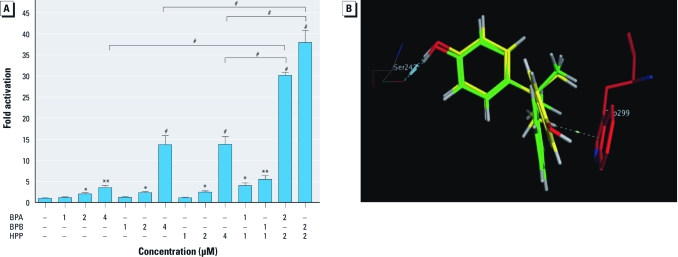
BPA and analogues BPB and HPP synergistically activate PXR. (*A*) Activation of PXR in HepG2 cells transfected with full-length hPXR together with CYP3A4-luc reporter and CMX–β-galactosidase plasmid, and then treated with BPA, BPB, HPP, or mixtures. Error bars indicate SEM. (*B*) Docking poses of BPA (yellow) and HPP (green) in the binding pocket of hPXR. BPA and HPP may maintain hydrogen bond with Ser247; however, the orientation of the benzene ring of HPP is better than that of BPA for π-stacking interaction toward Trp299 (red). *p < 0.05, **p < 0.01, and ^#^*p* < 0.001 compared with the untreated group or groups treated with a single compound (*n* = 3).

*BPA and analogues synergistically activate hPXR.* Because of the large and flexible LBD of PXR, combinations of BPA and other EDCs may activate hPXR in an additive or synergistic manner. To explore this possibility, we examined mixtures of BPA with each of the analogues under study at various concentrations. These experiments revealed that BPA and HPP can activate PXR synergistically (Figure 5A). A mixture of 2 μM BPA and 2 μM HPP activated PXR and induced PXR-mediated reporter activity > 30-fold, whereas BPA or HPP alone at 4 μM induced reporter activity only 4- and 13-fold, respectively. Mixtures of BPB and HPP synergistically activated PXR in a similar manner (Figure 5A). These results show that combinations of BPA-like agonists can work synergistically to activate hPXR. In contrast, DPP, which lacks PXR agonistic activity, failed to affect the agonistic activity of BPA, BPB, or HPP [Supplemental Material, Figure 3 (http://dx.doi.org/10.1289/ehp.1104426)].

In view of HPP’s agonistic activity, we computationally docked HPP in the PXR binding pocket [see Supplemental Material, Figure 4 (http://dx.doi.org/10.1289/ehp.1104426)]. HPP binding was similar to that of BPA, although with a more optimal interaction with Trp299. Regarding the observed synergistic agonist activity of combinations of BPA and HPP, computational alignment of their separate binding poses supported the possibility that both BPA and HPP may bind to hPXR simultaneously and still maintain hydrogen bonding interactions with Ser247 (Figure 5B).

**Figure 6:**
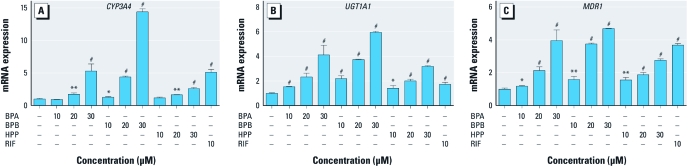
BPA and analogues regulate hPXR target gene expression. Human LS180 intestinal epithelial cells were treated with control medium (medium plus DMSO) or medium containing BPA, BPB, HPP, or RIF. Total RNA was isolated and gene expression levels were analyzed by QPCR with primers for human *CYP3A4* (*A*), *UGT1A1* (*B*), or *MDR1* (*C*). All values were normalized to *GAPDH* (glyceraldehyde-3-phosphate dehydrogenase) controls. Error bars indicate SEM. *p < 0.05, **p < 0.01, and ^#^*p* < 0.001 (*n* = 3 per treatment).

*BPA and analogues induce PXR target gene expression in human cells.* Because BPA and certain structural analogues are selective agonists for hPXR over mPXR, we used the human intestinal cell line LS180 to test the effect of BPA and analogues on PXR target genes. LS180 cells were treated with the solvent control, positive control (RIF), BPA, or an analogue of BPA (BPB or HPP) at various concentrations. Total RNA was isolated, and quantitative PCR (QPCR) was performed to detect the expression levels of PXR target genes involved in phase 1 [*CYP3A4* (cytochrome P450, family 3, subfamily A, polypeptide 4], phase 2 [*UGT1A1* (UDP glucuronosyltransferase 1 family, polypeptide A1)], and phase 3 [*MDR1* (multidrug resistance protein 1 or ATP-binding cassette, sub-family B (MDR/TAP), member 1)] metabolism. As shown in Figure 6, BPA significantly induced the expression of mRNA encoding the PXR target genes *CYP3A4*, *UGT1A1*, and *MDR1* in a dose-dependent manner compared with solvent controls. Consistent with the reporter assays, BPB and HPP also acted as PXR agonists and induced PXR target gene expression in LS180 cells.

## Discussion

Despite increasing evidence that exposure to BPA causes adverse health effects in humans, controversy remains about the specificity and mechanism of those effects (Vandenberg et al. 2009). Although BPA is a xenoestrogen, its endocrine-disrupting effects cannot be entirely attributed to its estrogenic activity. More mechanistic studies are urgently needed to explore BPA’s effects on other signaling pathways. Masuyama et al. (2000) reported that BPA has no effect on mPXR-mediated transcription. Later studies found that BPA can activate hPXR and induce PXR target genes in human primary cells (Tabb et al. 2004; Takeshita et al. 2001). In the present study, we sought to determine and explore the mechanism by which BPA interacts with and activates PXR. We found that BPA is a potent agonist for hPXR but not for mPXR and rPXR. We further identified the key residues within PXR’s ligand-binding pocket interacting with BPA by combining analyses of computational docking and site-directed mutagenesis. Several structural analogues of BPA, including BPB, HPP, and BPAF, were identified as hPXR agonists. Our findings suggest that hPXR is a target of BPA and certain analogues and that activation of PXR by BPA may contribute to BPA’s adverse effects in humans.

A fundamental question about all EDCs is whether low-dose exposure to EDCs can influence human endocrine functions. Here we present experimental results that show, for the first time, that BPA and specific analogues can synergistically activate hPXR. Although BPA activates hPXR at relatively high concentrations *in vitro* (~ 2 μM), combinations of BPA and numerous other EDCs may additively or synergistically activate hPXR *in vivo*. Additional *in vivo* studies are required to establish the influence of such synergistic or additive effects in risk assessment, because exposure to mixtures of chemicals is much more representative of real-world scenarios. If additive or synergistic effects are confirmed under *in vivo* conditions, then PXR-mediated effects of BPA, even at low doses, must be assessed within the context of the influence of coexisting EDCs.

The computational modeling studies, in concert with site-directed mutagenesis analysis, demonstrated their utility to predict, visualize, and quantify the binding of novel ligands of nuclear receptors at the molecular level. Among the series of structural analogues of BPA tested, several, including BPB and HPP, exhibited activity as hPXR agonists. The variability in PXR activity among these close structural analogues of BPA illustrates that even slight alteration of the BPA structure can translate to dramatic effects on the compound’s ability to activate PXR. Based on the docking studies and reporter assays, we deduce that an essential hydrogen bonding interaction with Ser247 and extensive hydrophobic contacts with receptor residues are structural requirements of BPA analogues that activate hPXR. The critical hydrogen bond with Ser247 localizes BPA into a hydrophobic region of the PXR ligand-binding pocket lined by Phe288, Trp299, and Tyr306. These three residues are highly conserved across species and interact with structurally diverse PXR agonists, as evidenced by multiple PXR crystal structures. Numerous hydrophobic contacts between BPA and PXR further stabilize ligand binding and indirectly promote contact with the AF-2 region, thus facilitating proper orientation of αAF to interact with coactivators.

Activation of hPXR by BPA may contribute to adverse effects and should be considered in future risk assessment studies of BPA in humans. For example, human BPA exposure has been positively associated with cardiovascular disease in epidemiological studies (Lang et al. 2008; Melzer et al. 2010). However, mechanisms that may be responsible for these associations remain largely unknown. To our knowledge, BPA has not been shown to have atherogenic effects in animal models. Recently, we have demonstrated PXR’s atherogenic effects in animal models (Sui et al. 2011; Zhou et al. 2009a). In addition, chronic activation of mPXR by PCN was reported to increase atherosclerosis in apolipoprotein E–deficient mice (Zhou et al. 2009a). Because BPA is an agonist for hPXR, chronic activation of hPXR by exposure to BPA may lead to increased atherosclerosis development and cardiovascular disease risk in humans.

## Conclusion

We have shown that BPA and several analogues are potent agonists for hPXR but not mPXR. Computational docking studies and site-directed mutagenesis analysis identified key residues within PXR’s ligand-binding pocket that interact with BPA. The BPA analogues BPB, HPP, and BPAF were also identified as hPXR agonists. Interestingly, combinations of BPA with certain agonistic analogues acted synergistically in activating PXR. The present study highlights the importance of the choice of animal model in the human risk assessment of BPA.

We hope that findings from this study will stimulate further investigations of BPA and its analogues, in particular the mechanism by which BPA activates hPXR, the species specificity of BPA activation of PXR, and the prospect of additive or synergistic effects of BPA and BPA-like agonists on hPXR in combination with each other or with other EDCS. Activation of PXR by BPA, alone or in combination with analogues or a wider array of EDCs, may explain at least some of the adverse health effects in humans.

## Supplemental Material

(225 KB) PDF
