# Supplementary material for: Bisphenol A and Its Analogues Activate Human Pregnane X Receptor
Source: Environ Health Perspect. 2012 Jan 3;120(3):399–405. doi: 10.1289/ehp.1104426 (PMC3295358; doi:10.1289/ehp.1104426)
Supplement: (225 KB) PDF [file ehp.1104426.s001.pdf]

Supplemental Material

Bisphenol A and Its Analogs Activate Human Pregnane X Receptor

Yipeng Sui[1], Ni Ai[2], Se-Hyung Park[1], Jennifer Rios-Pilier[1], Jordan T. Perkins[1], William J. Welsh[2], and Changcheng Zhou[1]

[1]Graduate Center for Nutritional Sciences, University of Kentucky, Lexington, Kentucky

[2]Department of Pharmacology, Robert Wood Johnson Medical School, University of Medicine and Dentistry of New Jersey, Piscataway, New Jersey

Table of Contents

Supplemental Material, Table 1.

Primer sequences for site-directed mutagenesis and QPCR p. 3

Supplemental Material, Figure 1.

BPA cytotoxicity in HepG2 cells p. 4

Supplemental Material, Figure 2.

BPA-glucuronide does not activate human PXR p. 5

Supplemental Material, Figure 3

DPP does not affect BPA and its analogs' agonistic activity p. 6

Supplemental Material, Figure 4

An interaction map of HPP and human PXR p. 7

Supplemental Material, Table 1.

Primer sequences for site-directed mutagenesis and QPCR.

Mutant	Primer sequences
PXR-S247L	5’-CTGCTGCCCCACATGGCTGACATGTTAACCTACATGTTCAAAGGC-3’
	5’-GCCTTTGAACATGTAGGTTAACATGTCAGCCATGTGGGGCAGCAG-3’
PXR-S247A	5’-CTGCTGCCCCACATGGCTGACATGGCAACCTACATGTTCAAAGGC-3’
	5’-GCCTTTGAACATGTAGGTTGCCATGTCAGCCATGTGGGGCAGCAG-3’
PXR-C284S	5’-GAAGGGGGCCGCTTTCGAGCTGAGTCAACTGAGATTCAACACAG-3’
	5’-CTGTGTTGAATCTCAGTTGACTCAGCTCGAAAGCGGCCCCCTTC-3’
PXR-Q285L	5’-GGGCCGCTTTCGAGCTGTGTCTACTGAGATTCAACACAGTG-3’
	5’-CACTGTGTTGAATCTCAGTAGACACAGCTCGAAAGCGGCCC-3’
PXR-F288A	5’-CGAGCTGTGTCAACTGAGAGCCAACACAGTGTTCAACGCGG-3’
	5’-CCGCGTTGAACACTGTGTTGGCTCTCAGTTGACACAGCTCG-3’
PXR-W299L	5’-CAACGCGGAGACTGGAACCTTGGAGTGTGGCCGGCTGTCC-3’
	5’-GGACAGCCGGCCACACTCCAAGGTTCCAGTCTCCGCGTTG-3’
PXR-Y306F	5’-GGGAGTGTGGCCGGCTGTCCTTCTGCTTGGAAGACACTGCAGG-3’
	5’-CCTGCAGTGTCTTCCAAGCAGAAGGACAGCCGGCCACACTCCC-3’
PXR-L308F	5’-GGCCGGCTGTCCTACTGCTTCGAAGACACTGCAGGTGGC-3’
	5’-GCCACCTGCAGTGTCTTCGAAGCAGTAGGACAGCCGGCC-3’
PXR-M323L	5’-CAGCAACTTCTACTGGAGCCCCTGCTGAAATTCCACTACATGCTG-3’
	5’-CAGCATGTAGTGGAATTTCAGCAGGGGCTCCAGTAGAAGTTGCTG-3’
PXR-L411F	5’-GCTCAGCACACCCAGCGGTTCCTGCGCATCCAGGACATAC-3’
	5’-GTATGTCCTGGATGCGCAGGAACCGCTGGGTGTGCTGAGC-3’
CYP3A4	5’-GGCTTCATCCAATGGACTGCATAAAT-3’
	5’-TCCCAAGTATAACACTCTACACAGACAA-3’
UGT1A1	5’-TGCTCATTGCCTTTTCACAG-3’
	5’-GGGCCTAGGGTAATCCTTCA-3’
MDR1	5’-CCCATCATTGCAATAGCAGG-3’
	5’-GAGCATACATATGTTCAAACTTC-3’
GAPDH	5’-GGCCTCCAAGGAGTAAGACC-3’
	5’-AGGGGAGATTCAGTGTGGTG-3’

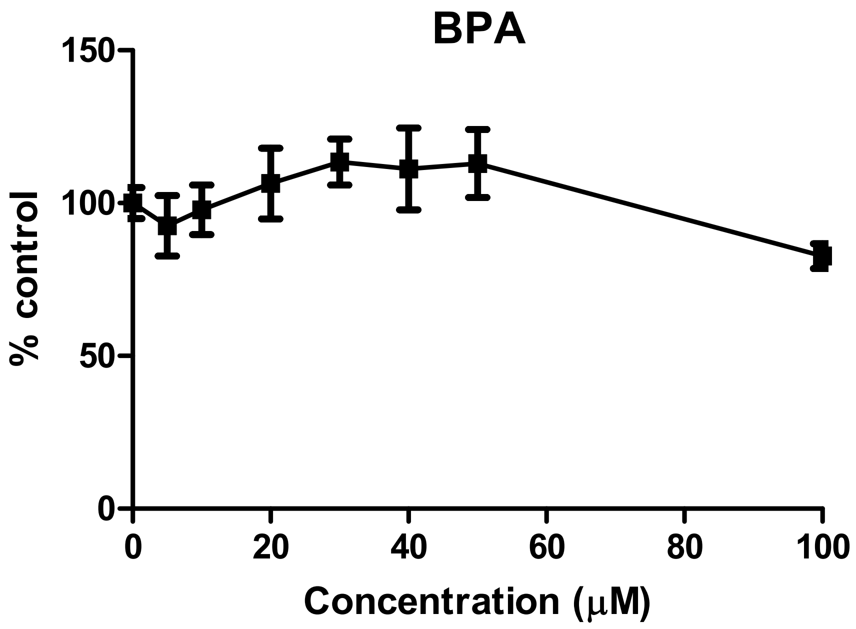

Supplemental Material, Figure 1. BPA cytotoxicity in HepG2 cells. HepG2 cells were seeded into 96 well plates and cultured for 24 h. The cells were treated with BPA at indicated concentrations for 24 h. 10 μl of 5 mg/ml MTT solution was added to each well and the cells were cultured for additional 3 h. Cell culture medium was then removed and 100 μl of DMSO was added into each well. Absorbance was measured at 570 nm and cell viability is expressed as percent viability compared to control treatment.

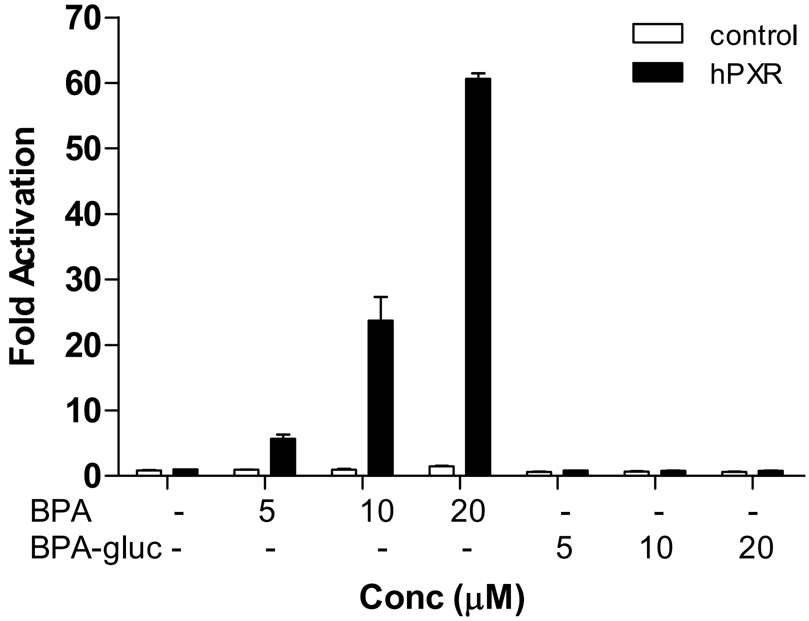

Supplemental Material, Figure 2. BPA-glucuronide does not activate human PXR. HepG2 cells were transfected with full-length hPXR together with CYP3A4-luc reporter and CMX-β-galactosidase control plasmid. Cells were treated with DMSO control, BPA, or BPA-glucuronide (BPA-gluc) at the indicated concentrations for 24 h.

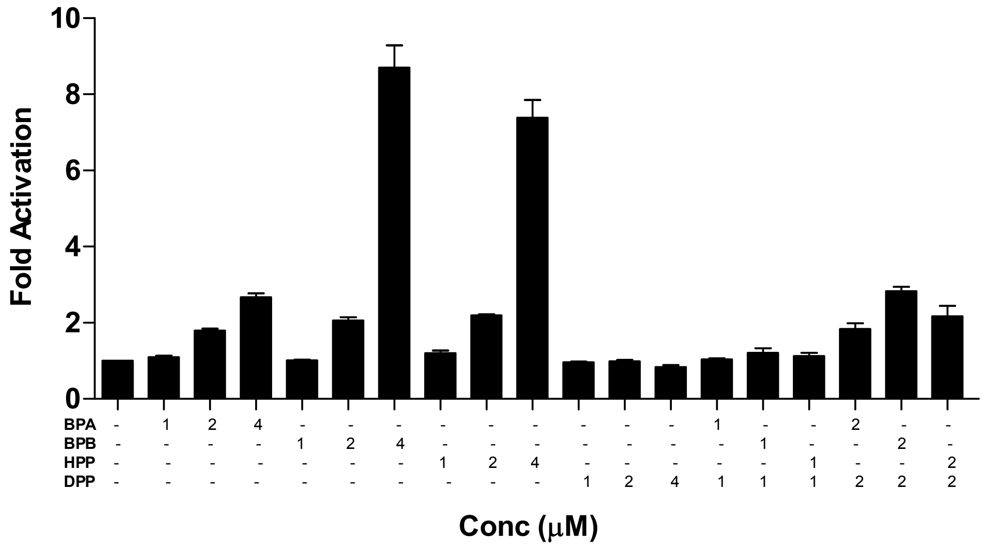

Supplemental Material, Figure 3. DPP does not affect BPA and its analogs' agonistic activity. HepG2 cells were transfected with full-length hPXR together with CYP3A4-luc reporter and CMX-β-galactosidase control plasmid. Cells were treated with BPA, BPB, HPP, DPP or mixtures at indicated concentrations.

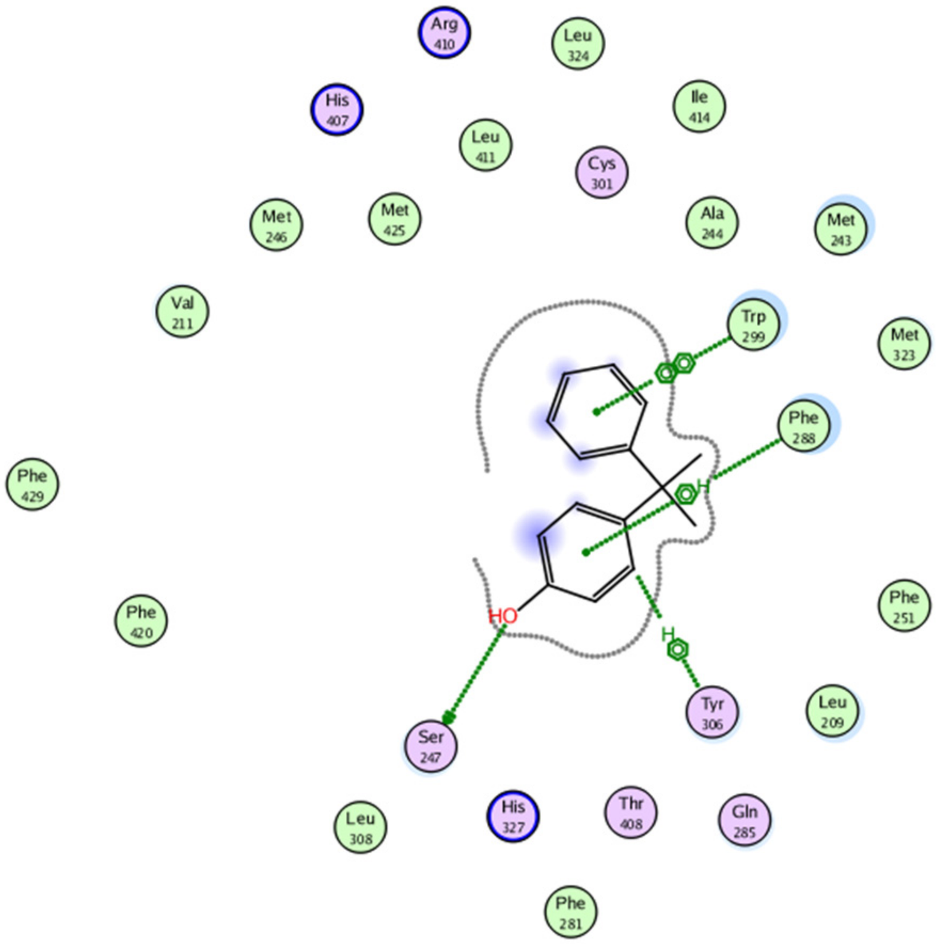

Supplemental Material, Figure 4. An interaction map of HPP and human PXR. HPP was computationally docked in the ligand binding pocket of the hPXR apoprotein X-ray crystal structure (3hvl.pdb). HPP binds to PXR binding pocket in a similar manner as BPA but has a better interaction with Trp299.
